# From IL-17 to IFN-γ in inflammatory skin disorders: Is transdifferentiation a potential treatment target?

**DOI:** 10.3389/fimmu.2022.932265

**Published:** 2022-07-28

**Authors:** Arno Belpaire, Nanja van Geel, Reinhart Speeckaert

**Affiliations:** Department of Head and Skin, Ghent University Hospital, Gent, Belgium

**Keywords:** Th17, IL-17, plasticity, IFN and y, inflammatory skin disease, Th17.1, psoriasis, vitiligo

## Abstract

The targeted inhibition of effector cytokines such as interleukin 17 (IL-17) in psoriasis and IL-13 in atopic dermatitis offers impressive efficacy with a favorable side effect profile. In contrast, the downregulation of interferon gamma (IFN-γ) in T helper (Th) 1-dominant skin disorders may lead to more adverse events, given the crucial role of IFN-γ in antiviral and antitumoral immunity. Modulating Th17 and Th2 cell differentiation is performed by blocking IL-23 and IL-4, respectively, whereas anti-IL-12 antibodies are only moderately effective in downregulating Th1 lymphocyte differentiation. Therefore, a targeted approach of IFN-γ-driven disorders remains challenging. Recent literature suggests that certain pathogenic Th17 cell subsets with Th1 characteristics, such as CD4^+^CD161^+^CCR6^+^CXCR3^+^IL-17^+^IFN-y^+^ (Th17.1) and CD4^+^CD161^+^CCR6^+^CXCR3^+^IL-17^-^IFN-y^+^ (exTh17), are important contributors in Th1-mediated autoimmunity. Differentiation to a Th17.1 or exTh17 profile results in the upregulation of IFN-y. Remarkably, these pathogenic Th17 cell subsets are resistant to glucocorticoid therapy and the dampening effect of regulatory T cells (Treg). The identification of Th17.1/exTh17 cells in auto-immune disorders may explain the frequent treatment failure of conventional immunosuppressants. In this review, we summarize the current evidence regarding the cellular plasticity of Th17 cells in inflammatory skin disorders. A deeper understanding of this phenomenon may lead to better insights into the pathogenesis of various skin diseases and the discovery of a potential new treatment target.

## 1 Introduction

Epithelial tissues harbor a substantial number of IL-17-producing immune cells as IL-17 is crucial for immune barrier protection. IL-17 protects against not only pathogens that are not adequately addressed by Th1 or Th2 immunity, such as fungi, but also gram-negative and gram-positive bacteria (1, 2). The IL-17 pathway creates a strong inflammatory response by upregulating a broad range of cytokines, neutrophil-recruiting chemokines, and antimicrobial peptides. Because of its critical role in barrier immunity and synergistic effect with other cytokines (*e*.*g*., TNF-, IFN-, and IL-1), IL-17 is an early contributor to a variety of skin disorders (2). Th17 cells are known key players in inflammatory skin diseases, such as psoriasis (3). More than a decade ago, it was assumed that each of the effector T cell subsets was in a fixed state after differentiation (4). More recent data indicate that particular cell subsets can acquire characteristics from other effector T cell subsets in response to the local microenvironment. Particularly, Th17 lymphocytes may acquire a Th1-like phenotype, resulting in the expression and production of IFN-y. This “functional plasticity” of CD4^+^CD161^+^ T cells plays a pivotal role in the pathogenesis of autoimmune diseases and offers a new perspective in the ongoing search for new treatment targets (5, 6). This review focuses on the current evidence of Th17 plasticity in inflammatory skin diseases and systemic diseases with cutaneous involvement.

## 2 Key mechanisms of functional cell plasticity

**Figure 1:**
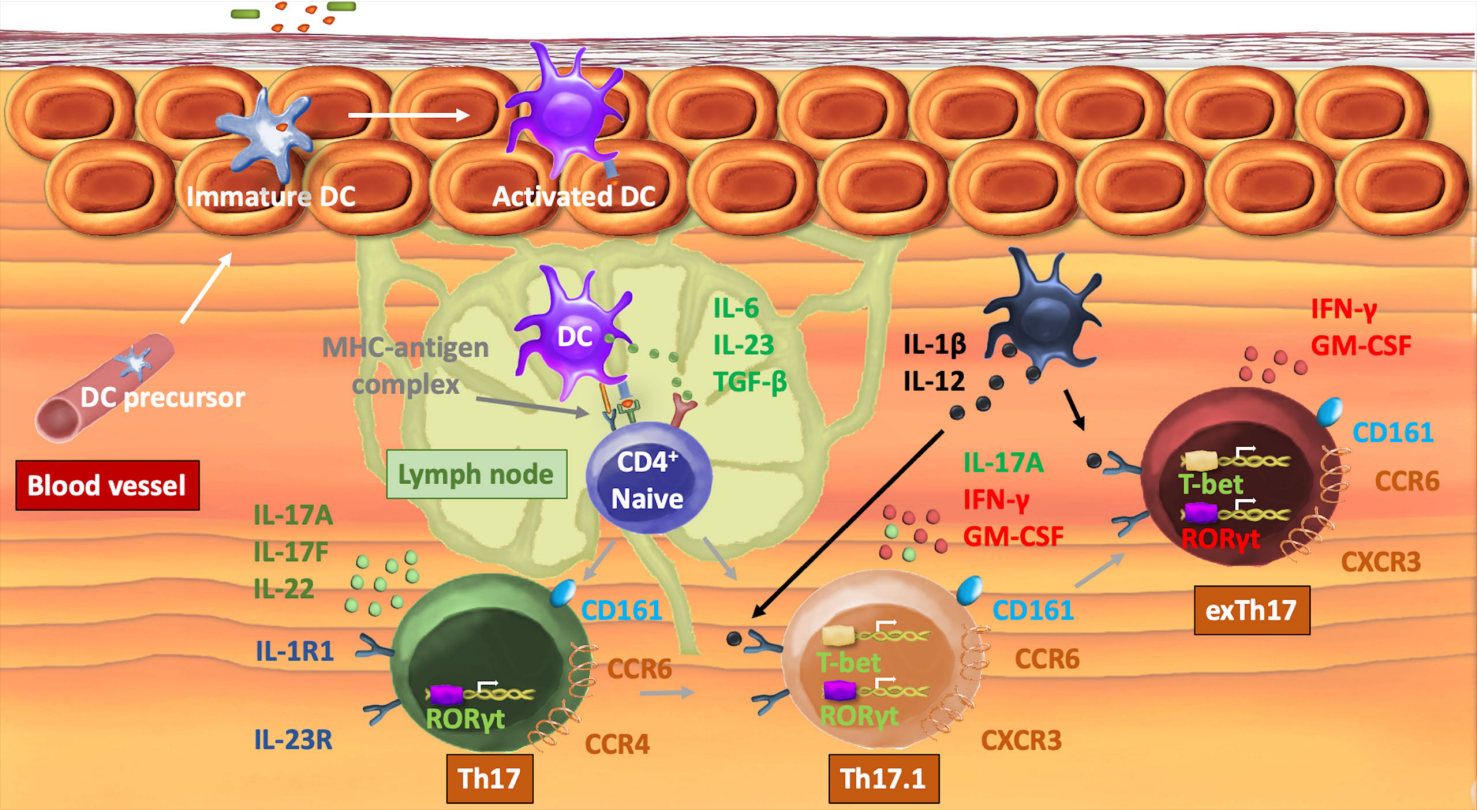
Mechanisms of Th17 plasticity. After recognition of an antigen, a DC translocates to a neighboring lymph node. Activation of a naive T-cell occurs by interaction of the MHC–antigen complex with the T-cell receptor. IL-6, IL-23, and TGF-β induce the expression of the transcription factor RORγt that orchestrates the differentiation of the Th17 lineage and directly induces the transcription of IL-17A/F and IL-22 as well as chemokine receptors CCR4 and CCR6. In the presence of pro-inflammatory cytokines IL-12 and IL-1β, T-bet is expressed, which enables transdifferentiation into a Th17.1 cell subset, characterized by the production of both IL-17 and IFN-γ as well as the expression of CXCR3. In specific circumstances, Th17 loses the capacity to produce IL-17 and becomes exTh17 cells. Th17, t-helper 17; DC, dendritic cell; CD, cluster of differentiation; MHC, major histocompatibility complex; IL, interleukin; TGF-β, transforming growth factor β; RORγt, retinoic acid receptor-related orphan nuclear receptor ɣt; C(X)CR, C(X)C chemokine receptor; T-bet, T-box protein expressed in T cells.

As depicted in **Figure 1**, Th17 differentiation is initiated by the presence of IL-6, transforming growth factor beta (TGF-β), and IL-23, subsequently activating the master transcription factor retinoid-related orphan receptor-γt (RORγt) and signal transducer and activator of transcription 3 (STAT3) (7, 8). Conventional Th17 cells (CD4^+^CD161^+^CCR6^+^IL17^+^IFN-y^-^) are then able to produce their signature cytokines interleukin (IL) 17A, IL-17F, and IL-22 (9). Elevated levels of pro-inflammatory cytokines, in particular, IL-12, induce a subset of Th17 cells, in which IFN-y production is upregulated by the activation of STAT4 (10, 11).. This newly defined Th17.1 (CD4^+^CD161^+^CCR6^+^CXCR3^+^IL-17^+^IFN-y^+^) subset shares phenotypic features from both Th17 and Th1 cell lineages and expresses both RORγt and T-box expressed in T cells (T-bet) (12). In addition to IFN-y, Th17.1 cells produce granulocyte–macrophage colony-stimulating factor and CCL20 (13). Pathogenic Th17 cells may completely lose the expression of IL-17 and differentiate into exTh17 (CD4^+^CD161^+^CCR6^+^IL17^+^IFN-y^+^). The regulation of the functional plasticity of Th17 cells occurs at different stages within the cell and is not yet fully understood. A comprehensive description of the molecular mechanisms, genetic profiling, and epigenetic modifications involved in cell plasticity has been reviewed elsewhere (14, 15).

### 2.1 Pathogenic Th17 lymphocytes in inflammatory skin diseases

#### 2.1.1 Vitiligo

Central to the disease process of vitiligo is the autoimmune destruction of melanocytes, in which IFN-y plays an important role (16). Although the pathogenic effect of Th17 cells in vitiligo is disputed, elevated levels of IL-17 in both blood and skin samples from vitiligo patients have been demonstrated in several studies (17, 18). The capacity of IL-17 to decrease melanogenesis is modest, but combined with IFN-γ and tumor necrosis factor alpha (TNF-α), there is a synergistic effect on pigmentation and inhibition of the function and survival of melanocytes (19). Nonetheless, IL-17 blockade fails to halt disease progression. Further analysis showed that Th17.1 cells are increased in vitiligo and are likely an important source of the elevated IL-17 concentrations (20). This was confirmed by another study revealing an impressive increase in CD4^+^CCR6^+^CXCR3^+^ T cells compared to those in stable patients and healthy controls. Interestingly, the Th17.1 levels decreased dramatically after treatment (21). The frequency of (peri-)lesional Th17.1 cells has not yet been investigated in progressive vitiligo patients. New cases of vitiligo have been reported in patients receiving ustekinumab and secukinumab (22). On the other hand, some patients with improvement have been documented with ustekinumab in case of concomitant psoriasis (23). Vitiligo exhibits a complex immune environment with a likely contribution of Th17 plasticity.

#### 2.1.2 Alopecia areata

Besides the increased IFN-γ levels, a meta-analysis of 10 studies revealed increased IL-17 levels in 9 out of 10 studies. The IL-23 concentrations were also higher in alopecia areata (AA) patients compared to healthy controls (24). Half of the infiltrating CD4+ T lymphocytes present around the hair follicles in AA were composed of the Th17 phenotype (25). However, the pathogenic role of IL-17 in AA remains a controversial topic. Similar to vitiligo, IL-17 inhibition did not have a significant effect on hair regrowth in AA (26). On the contrary, case reports have demonstrated new onset of AA during treatment with secukinumab (27). In addition, IL-12/23 inhibition in AA demonstrated variable results. In some cases, treatment with ustekinumab induced significant hair regrowth, whereas no improvement was observed in other patients (28–30). As both IL-12 and IL-23 drive Th17 cells towards a Th17.1 or exTh17 phenotype, the observed beneficial responses of IL-12/23 inhibition might be due to interference with the mechanisms that drive Th17 plasticity, although the lack of consistent outcomes in studies suggests that other cytokines are also involved.

#### 2.1.3 Psoriasis

The pathogenesis of psoriasis is characterized by a complex interplay between IL-17 and IFN-y producing CD4^+^ and CD8^+^ T-cell subsets (31). Before the identification of IL-17, an upregulation of the IL-12/IFN-γ signaling pathway was considered as the major driving disease mechanism in psoriasis since elevated IFN-γ levels were correlated with disease severity and were observed in serum and skin samples (both lesional and non-lesional) (32). Furthermore, IFN-γ-induced chemokines, such as CXCL9, CXCL10, and CXCL11, were upregulated in psoriatic lesions (33). A paradigm shift towards the IL-23/IL-17 axis as the central mechanism of the pro-inflammatory cycle of psoriasis has questioned the relevance of Th1 cells and IFN-y as the main drivers of the disease. At present, the exact role of IFN-y in relation to the IL-17/IL-23 axis is unclear (34, 35). Meanwhile, the recognition of resident memory T cells (Trm) in disease relapse and emerging evidence of IL-17^+^/IFN-y^+^ double-producing T-cell subsets (both CD4^+^ and CD8^+^) contribute to our understanding of the full disease mechanism (36, 37). Increased frequencies of Th17.1 cells in the dermis of psoriasis patients were already detected more than a decade ago (38). In 2009, Zaba et al. demonstrated that the levels of CD11c^+^ blood dendritic cell antigens (BDCA)-1^-^ DCs were increased 30-fold in psoriatic lesional skin compared to healthy skin. This DC population induced a T helper subset that produced both IFN-γ and IL-17. In contrast, CD11c^+^BDCA-1^+^ DCs, considered as the main dermal DC population in normal skin, and CD163^+^ macrophages were unable to induce this specific cell subset (39). In a recent article, the number of Th17 lymphocytes in peripheral blood samples of psoriasis patients significantly correlated with disease severity, although no correlation was detected for Th17.1 cells. Another study documented a non-significant increase of Th17.1 lymphocytes in psoriasis compared to healthy controls. Positive correlations between disease severity and lesional Th17 and Th17.1 cells were found. Treatment with etanercept significantly reduced the percentages of CD4^+^IL-17^+^IFN-y^-^ cells, while the percentages of CD4^+^IL17^+^IFN-y^+^ lymphocytes and CD4^+^IL-17^-^IFN-y^+^ cells remained unchanged (40). Although these data seem to indicate a limited contribution of Th17.1/exTh17 lymphocytes to the pathogenesis of psoriasis, the extent to which IFN-γ-producing Th17 subsets are involved in the inflammatory loop may depend on the psoriasis phenotype. Frequencies of circulating Th17.1 cells are significantly increased in patients with guttate psoriasis compared to plaque psoriasis and healthy control subjects. An explanation could be the decreased frequency of CD4^+^CD25^high^ Tregs in guttate psoriasis. CD4^+^CD25^high^ Tregs are capable of dampening the IFN-γ levels, but not the IL-17 levels. CD4^+^ T cells from patients with guttate psoriasis induce more apoptosis of keratinocytes and promote keratinocyte proliferation, which contributes to the initiation of the disease (41).

#### 2.1.4 Acne

The IL-17 levels are elevated in acne lesions. IL-6, IL-23, and TGF-β are highly expressed in addition to IL-17A, IL-22, IL-26, TNF-α as well as the chemokines CSF2 and CCL20. T-bet, CXCR3, and IFN-γ are also upregulated, indicating the contribution of Th1 effector cells in acne lesions. Additionally, the IFN-γ-induced chemokines—CXCL9, CXCL10, and CXCL11—are overexpressed (42). The combined expression of CXCR3 and CD161 was present in 15% of conventional T cells, reminiscent of pathogenic Th17.1 lymphocytes (43). *Cutibacterium acnes* can trigger the concomitant production of IL-17 and IFN-γ (44). Peripheral mononuclear blood cells (PBMC) exposed to *Propionibacterium acnes* produce IL-1β, IL-6, IL-12, and IL-23, which polarizes T cells to acquire a Th1 and Th17 phenotype. *P. acnes*-reactive Th17.1 cells were induced in PBMCs of all donors, whereas Th1-like lymphocytes were only found in 40%. The inhibition of IL-1β decreased the percentages of Th17 and Th17.1 lymphocytes, whereas IL-12/IL-23 inhibition was only able to decrease the Th17.1 cells. Blocking both IL-1β and IL-12/23 resulted in superior results. *In vitro*, *P. acnes* or *Staphylococcus aureus* are only able to increase the Th17 and Th17.1 cells, but not CD4^+^IL-17^-^IFN-y^+^ lymphocytes. Patients with acne were much more responsive to *P. acnes* stimulation compared to healthy controls, whereas no difference was found after stimulation with *S. aureus* (44). These results indicate that *P. acnes* facilitates the development of Th17.1 lymphocytes without further transitioning into exTh17 lymphocytes.

#### 2.1.5 Hidradenitis suppurativa

Hidradenitis suppurativa (HS) displays a clustering of Th1/Th17-related cytokines based on messenger ribonucleic acid (mRNA) analysis of lesional skin. IFN-γ, IL-12, IL-17, and TNF-α are directly correlated with disease severity (45). A trend towards an increase in exTh17 lymphocytes was found in both skin and blood samples in HS patients, although the sample size was too small to demonstrate a significant correlation (46). CD4^+^ T cells in lesional skin produce similar amounts of IL-17 compared to psoriasis (47). Similar to acne, these findings point to a strong activated Th17 pathway, but without a pronounced evolution towards Th17.1 or exTh17 cells as found in Th1-mediated disorders. Interestingly, in patients suffering from both Crohn’s disease and HS, CD4^+^CD161^+^ T cells were found in perianal fistulae as well as HS lesions, indicating a possible association between both diseases with potential new therapeutic implications (48).

#### 2.1.6 Atopic dermatitis

Th17-related cytokines seem to contribute less to the inflammatory process of atopic dermatitis (AD) (49). A remarkable finding is the different phenotypic forms of AD depending on ethnicity, with a higher dominance of the Th17 axis seen in an Asian population (50). Interestingly, in related Th2-mediated conditions, such as chronic allergic asthma, IL-17-producing Th2 cells (CD4^+^CCR6^+^CRTH2^+^) have been induced in mouse models (51). Furthermore, allergen-specific Th2 lymphocytes can switch to IFN-γ-producing cells *in vitro*. IL-4+IFN-y+ “Th2.1” cells can also occur naturally in virus-infected mice (52). These observations point to a striking heterogeneity of different T cell subsets in atopic diseases, but evidence of CD4^+^IL17^+^ cells in AD patients is relatively scarce (53). In a study with Japanese AD patients, a decrease in both Th17 and Th17.1 cells was found, but only a reduction in Th17 cells was significant. The serum levels of CCL-17 and immunoglobulin E (IgE) and the number of eosinophils were negatively correlated with Th17 lymphocytes (54). In European AD patients, both Th17 and Th17.1 subsets were equally decreased (40). Other studies confirmed a decreased number of Th17 cells in the skin of AD patients (55). Similar to psoriasis, the contribution of Th17.1 to the inflammatory response may depend on the disease phenotype. Early-onset pediatric AD has higher IL-17 levels compared to adults with AD, with increased IFN-y in lesional *versus* non-lesional skin (56). In AD, a broad epidermal expression of endothelin-1 can be found, especially in chronic lesions. Endothelin-1 induces IL-12 and IL-23 production by dendritic cells which signal the downstream expression of IL-17, IL-22, and IFN-y (57).

### 2.2 Pathogenic Th17 lymphocytes in systemic diseases with cutaneous involvement

#### 2.2.1 Sarcoidosis

The role of Th17 lymphocytes in the pathogenesis of sarcoidosis has been extensively documented, with increased numbers of Th17 cells as well as an upregulation of IL-17 expression in peripheral blood, bronchoalveolar lavage fluid (BAL) as well as lung tissue and lymph nodes (58, 59). Multiple studies demonstrated higher numbers of Th17.1 cells in BAL fluid, peripheral blood, and lymph nodal aspirates from patients with sarcoidosis compared to a healthy control population. A greater increase of Th17.1 lymphocytes was seen in lymph nodal tissue and BAL fluid than in peripheral blood (60, 61). Remarkably, some authors have shown that elevated Th17.1 cells were mainly observed in the more favorable disease phenotypes of sarcoidosis, which has raised the question of whether other Th17 subsets may also exert a protective role (62). However, the development of sarcoidosis due to checkpoint inhibitors [anti-programmed death-ligand 1 (PD-L1) immunotherapy] is associated with a higher number of circulating Th17.1 cells at baseline. In addition, Arger et al. demonstrated that the frequency of Th17.1 lymphocytes increased with disease progression and when multiple organs were affected (63). This suggests that Th17.1 lymphocytes are activated during immunomodulating therapy and have a pathogenic role (64). The presence of Th17.1 cells has so far mainly been demonstrated in the lungs and lymph nodes of sarcoidosis patients. It seems plausible that similar cell subsets can also be found in other affected tissues, such as eyes and skin, as enhanced transcriptions of IL-12, IL-23, and IFN-γ have been observed in sarcoid skin lesions (65).

#### 2.2.2 Systemic lupus erythematosus

An increased number of Th17 lymphocytes and elevated levels of IL-17 have been demonstrated both in blood and affected tissue of patients with systemic lupus erythematosus (SLE) (66). Th17.1 cells are significantly expanded in SLE patients compared to healthy controls and correlate with disease activity. The number of Th17.1 lymphocytes is significantly higher in anti-DNA^+^ compared to anti-DNA^-^ SLE patients, although this is due to an overall increase in Th17 cells. The increase in Th17 cells may be due to the activation of the nucleotide-binding oligomerization domain, leucine-rich repeat, and pyrin-domain-containing 3 (NLRP3) by anti-DNA, thus promoting Th17 differentiation. In anti-DNA^+^ SLE patients, Th17.1 cells correlated negatively with complement 3 protein (67). These findings support a driving role of Th17 plasticity in lupus.

#### 2.2.3 Scleroderma

Pathogenic Th17 cell subsets are likely to contribute to skin fibrosis. The frequency of Th17.1 lymphocytes is increased both in the skin and in the circulation of patients with systemic scleroderma (68). A correlation with disease duration and severity was found. *In vitro* experiments showed that Th17.1 lymphocytes promoted the proliferation of fibroblasts and their capacity to produce collagen. The profibrotic function of Th17.1 lymphocytes can be attributed to the production of IL-21, as the inhibition of this cytokine decreased the levels of alpha smooth muscle actin and alpha-1 type I collagen mRNAs induced by Th17.1 cells (68).

#### 2.2.4 Graft *versus* host disease

Striking differences in cytokine signaling were observed between various subtypes of cutaneous graft *versus* host disease (GvHD). Acute GvHD displays a Th2 signature with an increased expression of IL-4, IL-5, and IL-13, but not IL-17 (69). In cutaneous psoriasiform GvHD, almost half of the Th17 cells were identified as Th17.1 cells (2.1% of total CD4^+^ cells). In chronic lichenoid GvHD, no Th17, Th17.1, or exTh17 lymphocytes were present, although Tc17 lymphocytes were detected (70). Other reports found a mixed Th1/Th17 signature in chronic lichenoid cGVHD. Mice experiments revealed that the expression of PD-L1 by host tissues suppresses the proliferation of Th17.1 cells. However, the synthetic retinoid Am80 restores the suppression of Th17.1 cell expansion due to low PD-L1 levels (71). Am80 is a retinoic acid receptor (RAR) α and RARβ-specific synthetic retinoid with more than 10-fold stronger activity compared to all-trans-retinoic acid. Am80 downregulates Th1 and Th17 differentiation and inhibits IFN-γ, IL-17, and TGF-β (72). These mouse experiments demonstrate that the functional plasticity of Th17 lymphocytes can be targeted both *in vitro* and *in vivo*, providing a promising proof-of-concept for future treatments.

## 3 Concluding remarks

**Figure 2:**
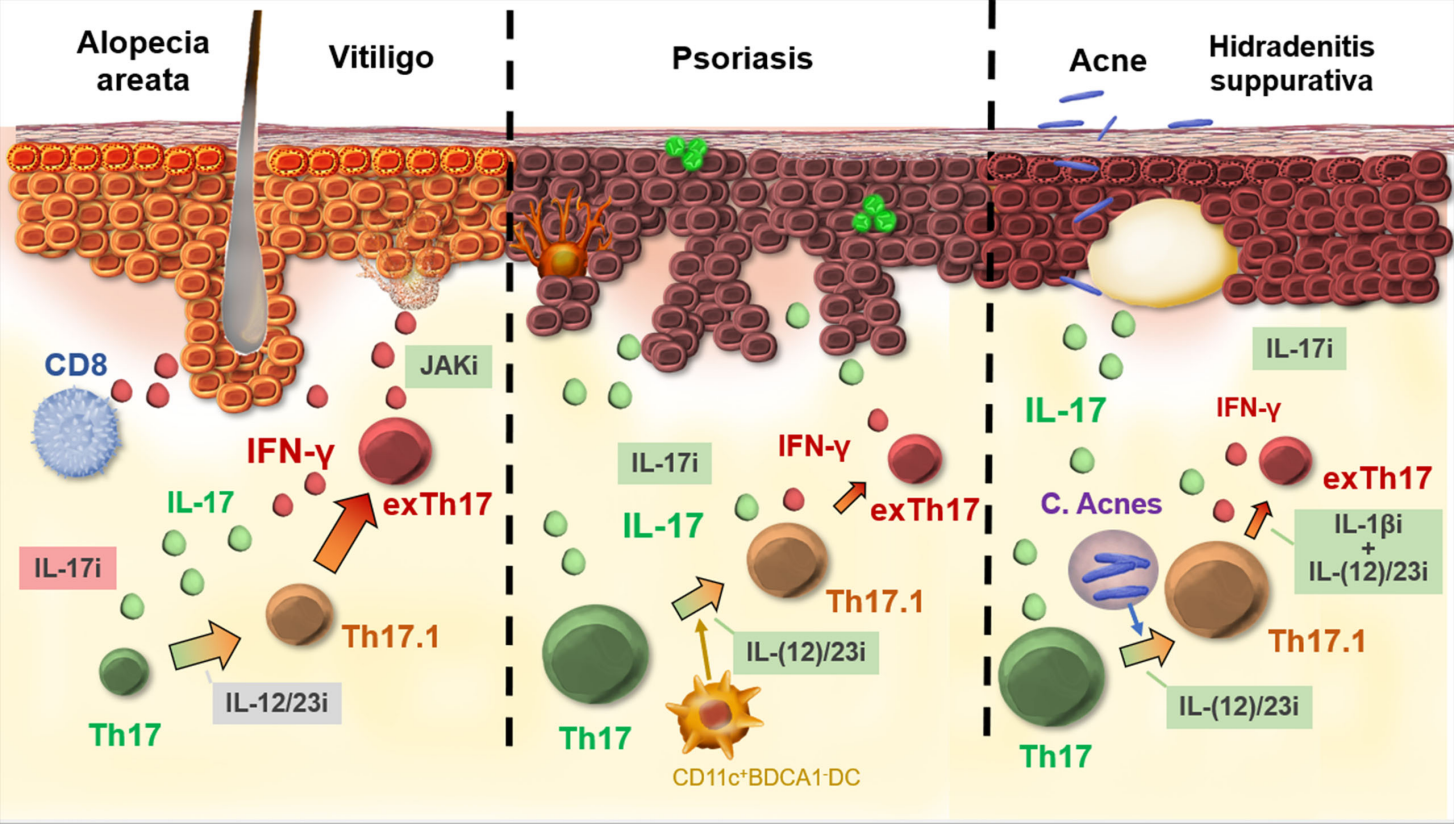
Th17 plasticity in skin diseases. In the case of IFN-γ-driven diseases, such as vitiligo and alopecia areata, full transdifferentiation from Th17 to exTh17 is likely. Biologics acting on Th17 and IL-17 fail to show efficacy for these disorders. In psoriasis, Th17.1 cells are not uncommon, although exTh17 cells are less important, as illustrated by the high efficacy of biologics acting on Th17/IL-17 and early transdifferentiation [*e*.*g*., IL-12/23 inhibition (i)]. Acne stimulates the formation of Th17.1, but exTh17 lymphocytes are less strongly induced. A combination treatment (IL-1βi anti-IL12/23i) is necessary to block transdifferentiation from Th17.1 to exTh17. Green boxes, good efficacy; gray boxes, variable efficacy; red boxes, no efficacy.

The development and the use of biologicals that act on the IL-23/IL-17 axis were an important turning point in the treatment of psoriasis (73). The spectacular therapeutic outcomes then raised the question of whether a similar effect could be achieved in other inflammatory skin diseases. However, in several Th1-dominant skin disorders such as alopecia areata and vitiligo, where increased IL-17 levels have also been documented, this targeted approach failed to induce an acceptable clinical response (20, 26). These observations suggest that IL-17 does not play a direct key role in driving Th1-dominant skin disorders. However, recent data has shown that pathogenic Th17 cell subsets with a more aggressive phenotype contribute to the production of IFN-y and thus may sustain or worsen the progression of IFN-y mediated skin diseases (74). This functional plasticity of Th17 cells is likely an underrecognized phenomenon, especially in disorders with high levels of IFN-γ (**Figure 2**). Dual IL-17+IFN-γ+ lymphocytes can further transdifferentiate into non-classical Th1 cells. ExTh17 cells are not constrained by Tregs and are more resistant to glucocorticoid suppression, which suggest that adapted therapeutic approaches may be necessary to block their pathogenic effects (75).

In IL-17-dominant skin disorders such as psoriasis, Th17.1/exTh17 are present, although less pronounced compared to IFN-γ-dominant skin disorders, and their inhibition seems not essential as demonstrated by the high efficacy of IL-17 inhibitors. In psoriasis, Th17 plasticity is present, especially in psoriasis guttata (41). Interestingly, in acne and hidradenitis suppurativa, mice experiments have shown the specific contribution of *C. acnes* in the development of dual IL-17^+^IFN-γ^+^ CD4^+^ cells, although in these disorders the subsequent transdifferentiation into IL-17^-^IFN-γ ^+^ exTh17 cells seems less pronounced (44, 46). The added value of targeting Th17 plasticity is currently still unclear for acne and hidradenitis suppurativa. Although the effects of Th17.1/exTh17 lymphocytes in Th2-mediated disorders such as AD seems negligible, there is evidence that the Th2 lineage is also more plastic than originally assumed (53). Regarding systemic disorders, substantial data on Th17 plasticity has been gathered in sarcoidosis, SLE, scleroderma, and GvHD (62, 68, 70, 76). Overall, Th17 plasticity is likely an underrecognized phenomenon, especially in disorders with high levels of IFN-γ and in case of skin fibrosis.

Cosmi et al. demonstrated that the transdifferentiation of T cells can be blocked by therapeutic intervention with biologicals (40). The idea of such a targeted approach is promising, but more focused research into the inducing cytokines, (epi)genetic modifications, and regulatory mechanisms that determine the development and behavior of transdifferentiated Th17 subsets in skin diseases remains to be done.

## Author contributions

In collaboration, RS and AB both carried out a literature search, both created the attached figures, and both drafted the manuscript. NG reviewed the manuscript and commented on the draft. All authors contributed to the article and approved the submitted version.

## Funding

FWO: Fundamental Clinical Mandate: 18B2721N The fundamental clinical mandate supports well-trained physicians and researchers who are pursuing a full-fledged career within translational research. The mandates offer the possibility of part-time release from a clinical position in function of the research. Translational research is the link between basic biomedical research, of capital importance to any progress, and clinical research, which focuses on patients. BOF Starting grant: Through the Starting Grant, financial support is granted for personnel, operating and/or equipment costs related to research. The budget is adequate to appoint 1 doctoral researcher (bursary) without seniority for a period of 4 years.

## Conflict of interest

The authors declare that the research was conducted in the absence of any commercial or financial relationships that could be construed as a potential conflict of interest.

## Publisher’s note

All claims expressed in this article are solely those of the authors and do not necessarily represent those of their affiliated organizations, or those of the publisher, the editors and the reviewers. Any product that may be evaluated in this article, or claim that may be made by its manufacturer, is not guaranteed or endorsed by the publisher.

## References

[1] Marks BR Craft J. Barrier immunity and IL-17. Semin Immunol (2009) 21(3):164–71. doi: 10.1016/j.smim.2009.03.001 PMC2692766 19386512

[2] Speeckaert R Lambert J Grine L Van Gele M De Schepper S van Geel N. The many faces of interleukin-17 in inflammatory skin diseases. Br J Dermatol (2016) 175(5):892–901. doi: 10.1111/bjd.14703 27117954

[3] Li B Huang L Lv P Li X Liu G Chen Y. The role of Th17 cells in psoriasis. Immunol Res (2020) 68(5):296–309. doi: 10.1007/s12026-020-09149-1 32827097

[4] Ruterbusch M Pruner KB Shehata L Pepper M. *In vivo* CD4(+) T cell differentiation and function: Revisiting the Th1/Th2 paradigm. Annu Rev Immunol (2020) 38:705–25. doi: 10.1146/annurev-immunol-103019-085803 32340571

[5] Misra DP Agarwal V. Th17.1 lymphocytes: emerging players in the orchestra of immune-mediated inflammatory diseases. Clin Rheumatol (2022). doi: 10.1007/s10067-022-06202-2 35546376

[6] DuPage M Bluestone JA. Harnessing the plasticity of CD4(+) T cells to treat immune-mediated disease. Nat Rev Immunol (2016) 16(3):149–63. doi: 10.1038/nri.2015.18 26875830

[7] Sallusto F. Heterogeneity of human CD4(+) T cells against microbes. Annu Rev Immunol (2016) 34:317–34. doi: 10.1146/annurev-immunol-032414-112056 27168241

[8] Saravia J Chapman NM Chi H. Helper T cell differentiation. Cell Mol Immunol (2019) 16(7):634–43. doi: 10.1038/s41423-019-0220-6 PMC6804569 30867582

[9] Littman DR Rudensky AY. Th17 and regulatory T cells in mediating and restraining inflammation. Cell (2010) 140(6):845–58. doi: 10.1016/j.cell.2010.02.021 20303875

[10] Zielinski CE Mele F Aschenbrenner D Jarrossay D Ronchi F Gattorno M. Pathogen-induced human TH17 cells produce IFN-gamma or IL-10 and are regulated by IL-1beta. Nature (2012) 484(7395):514–8. doi: 10.1038/nature10957 22466287

[11] Volpe E Servant N Zollinger R Bogiatzi SI Hupe P Barillot E. A critical function for transforming growth factor-beta, interleukin 23 and proinflammatory cytokines in driving and modulating human T(H)-17 responses. Nat Immunol (2008) 9(6):650–7. doi: 10.1038/ni.1613 18454150

[12] Maggi L Santarlasci V Capone M Rossi MC Querci V Mazzoni A. Distinctive features of classic and nonclassic (Th17 derived) human Th1 cells. Eur J Immunol (2012) 42(12):3180–8. doi: 10.1002/eji.201242648 22965818

[13] Noster R Riedel R Mashreghi MF Radbruch H Harms L Haftmann C. IL-17 and GM-CSF expression are antagonistically regulated by human T helper cells. Sci Transl Med (2014) 6(241):241ra80. doi: 10.1126/scitranslmed.3008706 24944195

[14] Cerboni S Gehrmann U Preite S Mitra S. Cytokine-regulated Th17 plasticity in human health and diseases. Immunology (2021) 163(1):3–18. doi: 10.1111/imm.13280 33064842 PMC8044333

[15] Stadhouders R Lubberts E Hendriks RW. A cellular and molecular view of T helper 17 cell plasticity in autoimmunity. J Autoimmun (2018) 87:1–15. doi: 10.1016/j.jaut.2017.12.007 29275836

[16] Bergqvist C Ezzedine K. Vitiligo: A review. Dermatology (2020) 236(6):571–92. doi: 10.1159/000506103 32155629

[17] Wang CQ Cruz-Inigo AE Fuentes-Duculan J Moussai D Gulati N Sullivan-Whalen M. Th17 cells and activated dendritic cells are increased in vitiligo lesions. PLoS One (2011) 6(4):e18907. doi: 10.1371/journal.pone.0018907 21541348 PMC3081835

[18] Zhou L Shi YL Li K Hamzavi I Gao TW Huggins RH. Increased circulating Th17 cells and elevated serum levels of TGF-beta and IL-21 are correlated with human non-segmental vitiligo development. Pigment Cell Melanoma Res (2015) 28(3):324–9. doi: 10.1111/pcmr.12355 25604047

[19] Zhou J An X Dong J Wang Y Zhong H Duan L. IL-17 induces cellular stress microenvironment of melanocytes to promote autophagic cell apoptosis in vitiligo. FASEB J (2018) 32(9):4899–916. doi: 10.1096/fj.201701242RR 29613836

[20] Speeckaert R Mylle S van Geel N. IL-17A is not a treatment target in progressive vitiligo. Pigment Cell Melanoma Res (2019) 32(6):842–7. doi: 10.1111/pcmr.12789 31063266

[21] Zhang L Kang Y Chen S Wang L Jiang M Xiang L. Circulating CCL20: A potential biomarker for active vitiligo together with the number of Th1/17 cells. J Dermatol Sci (2019) 93(2):92–100. doi: 10.1016/j.jdermsci.2018.12.005 30655106

[22] Mery-Bossard L Bagny K Chaby G Khemis A Maccari F Marotte H. New-onset vitiligo and progression of pre-existing vitiligo during treatment with biological agents in chronic inflammatory diseases. J Eur Acad Dermatol Venereol (2017) 31(1):181–6. doi: 10.1111/jdv.13759 27291924

[23] Elkady A Bonomo L Amir Y Vekaria AS Guttman-Yassky E. Effective use of ustekinumab in a patient with concomitant psoriasis, vitiligo, and alopecia areata. JAAD Case Rep (2017) 3(6):477–9. doi: 10.1016/j.jdcr.2017.07.009 PMC5614637 28971137

[24] Chang HC Lin MH Tsai HH. Serum levels of interleukin-17 and 23 in patients with alopecia areata: a systematic review and meta-analysis. Eur J Dermatol (2020) 30:200–1. doi: 10.1684/ejd.2020.3742 32293566

[25] Tanemura A Oiso N Nakano M Itoi S Kawada A Katayama I. Alopecia areata: infiltration of Th17 cells in the dermis, particularly around hair follicles. Dermatology. (2013) 226(4):333–6. doi: 10.1159/000350933 23838575

[26] Guttman-Yassky E Nia JK Hashim PW Mansouri Y Alia E Taliercio M. Efficacy and safety of secukinumab treatment in adults with extensive alopecia areata. Arch Dermatol Res (2018) 310(8):607–14. doi: 10.1007/s00403-018-1853-5 30121698

[27] Yalici Armagan B Atakan N. New onset alopecia areata during secukinumab therapy. Dermatol Ther (2019) 32(5):e13071. doi: 10.1111/dth.13071 31442356

[28] Guttman-Yassky E Ungar B Noda S Suprun M Shroff A Dutt R. Extensive alopecia areata is reversed by IL-12/IL-23p40 cytokine antagonism. J Allergy Clin Immunol (2016) 137(1):301–4. doi: 10.1016/j.jaci.2015.11.001 26607705

[29] Aleisa A Lim Y Gordon S Her MJ Zancanaro P Abudu M. Response to ustekinumab in three pediatric patients with alopecia areata. Pediatr Dermatol (2019) 36(1):e44–e5. doi: 10.1111/pde.13699 30338558

[30] Ortolan LS Kim SR Crotts S Liu LY Craiglow BG Wambier C. IL-12/IL-23 neutralization is ineffective for alopecia areata in mice and humans. J Allergy Clin Immunol (2019) 144(6):1731–4.e1. doi: 10.1016/j.jaci.2019.08.014 31470035 PMC6900443

[31] Casciano F Pigatto PD Secchiero P Gambari R Reali E. T Cell hierarchy in the pathogenesis of psoriasis and associated cardiovascular comorbidities. Front Immunol (2018) 9:1390. doi: 10.3389/fimmu.2018.01390 29971067 PMC6018171

[32] Chiricozzi A Romanelli P Volpe E Borsellino G Romanelli M. Scanning the immunopathogenesis of psoriasis. Int J Mol Sci (2018) 19(1):179. doi: 10.3390/ijms19010179 PMC5796128 29316717

[33] Kryczek I Bruce AT Gudjonsson JE Johnston A Aphale A Vatan L. Induction of IL-17+ T cell trafficking and development by IFN-gamma: mechanism and pathological relevance in psoriasis. J Immunol (2008) 181(7):4733–41. doi: 10.4049/jimmunol.181.7.4733 PMC2677162 18802076

[34] Lowes MA Russell CB Martin DA Towne JE Krueger JG. The IL-23/T17 pathogenic axis in psoriasis is amplified by keratinocyte responses. Trends Immunol (2013) 34(4):174–81. doi: 10.1016/j.it.2012.11.005 PMC3721313 23291100

[35] Harden JL Johnson-Huang LM Chamian MF Lee E Pearce T Leonardi CL. Humanized anti-IFN-gamma (HuZAF) in the treatment of psoriasis. J Allergy Clin Immunol (2015) 135(2):553–6. doi: 10.1016/j.jaci.2014.05.046 25085340

[36] Gallais Serezal I Hoffer E Ignatov B Martini E Zitti B Ehrstrom M. A skewed pool of resident T cells triggers psoriasis-associated tissue responses in never-lesional skin from patients with psoriasis. J Allergy Clin Immunol (2019) 143(4):1444–54. doi: 10.1016/j.jaci.2018.08.048 30268387

[37] Cosmi L Liotta F Maggi E Romagnani S Annunziato F. Th17 and non-classic Th1 cells in chronic inflammatory disorders: two sides of the same coin. Int Arch Allergy Immunol (2014) 164(3):171–7. doi: 10.1159/000363502 25033972

[38] Lowes MA Kikuchi T Fuentes-Duculan J Cardinale I Zaba LC Haider AS. Psoriasis vulgaris lesions contain discrete populations of Th1 and Th17 T cells. J Invest Dermatol (2008) 128(5):1207–11. doi: 10.1038/sj.jid.5701213 18200064

[39] Zaba LC Fuentes-Duculan J Eungdamrong NJ Abello MV Novitskaya I Pierson KC. Psoriasis is characterized by accumulation of immunostimulatory and Th1/Th17 cell-polarizing myeloid dendritic cells. J Invest Dermatol (2009) 129(1):79–88. doi: 10.1038/jid.2008.194 18633443 PMC2701224

[40] Antiga E Volpi W Cardilicchia E Maggi L Fili L Manuelli C. Etanercept downregulates the Th17 pathway and decreases the IL-17+/IL-10+ cell ratio in patients with psoriasis vulgaris. J Clin Immunol (2012) 32(6):1221–32. doi: 10.1007/s10875-012-9716-x 22699761

[41] Yan K Han L Deng H Fang X Zhang Z Huang G. The distinct role and regulatory mechanism of IL-17 and IFN-gamma in the initiation and development of plaque vs guttate psoriasis. J Dermatol Sci (2018) 92(1):106–13. doi: 10.1016/j.jdermsci.2018.07.001 30072243

[42] Kelhala HL Palatsi R Fyhrquist N Lehtimaki S Vayrynen JP Kallioinen M. IL-17/Th17 pathway is activated in acne lesions. PLoS One (2014) 9(8):e105238. doi: 10.1371/journal.pone.0105238 25153527 PMC4143215

[43] Eliasse Y Leveque E Garidou L Battut L McKenzie B Nocera T. IL-17(+) mast Cell/T helper cell axis in the early stages of acne. Front Immunol (2021) 12:740540. doi: 10.3389/fimmu.2021.740540 34650562 PMC8506309

[44] Kistowska M Meier B Proust T Feldmeyer L Cozzio A Kuendig T. Propionibacterium acnes promotes Th17 and Th17/Th1 responses in acne patients. J Invest Dermatol (2015) 135(1):110–8. doi: 10.1038/jid.2014.290 25010142

[45] Thomi R Cazzaniga S Seyed Jafari SM Schlapbach C Hunger RE. Association of hidradenitis suppurativa with T helper 1/T helper 17 phenotypes: A semantic map analysis. JAMA Dermatol (2018) 154(5):592–5. doi: 10.1001/jamadermatol.2018.0141 PMC6128497 29617527

[46] Moran B Sweeney CM Hughes R Malara A Kirthi S Tobin AM. Hidradenitis suppurativa is characterized by dysregulation of the Th17:Treg cell axis, which is corrected by anti-TNF therapy. J Invest Dermatol (2017) 137(11):2389–95. doi: 10.1016/j.jid.2017.05.033 28652108

[47] Lowe MM Naik HB Clancy S Pauli M Smith KM Bi Y. Immunopathogenesis of hidradenitis suppurativa and response to anti-TNF-alpha therapy. JCI Insight (2020) 5(19):5e139932. doi: 10.1172/jci.insight.139932 PMC7566733 32841223

[48] Giudici F Maggi L Santi R Cosmi L Annunziato F Nesi G. Perianal crohn's disease and hidradenitis suppurativa: a possible common immunological scenario. Clin Mol Allergy (2015) 13(1):12. doi: 10.1186/s12948-015-0018-8 26203298 PMC4511252

[49] Sugaya M. The role of Th17-related cytokines in atopic dermatitis. Int J Mol Sci (2020) 21(4):1314. doi: 10.3390/ijms21041314 PMC7072946 32075269

[50] Noda S Suarez-Farinas M Ungar B Kim SJ de Guzman Strong C Xu H. The Asian atopic dermatitis phenotype combines features of atopic dermatitis and psoriasis with increased TH17 polarization. J Allergy Clin Immunol (2015) 136(5):1254–64. doi: 10.1016/j.jaci.2015.08.015 26428954

[51] Wang YH Voo KS Liu B Chen CY Uygungil B Spoede W. A novel subset of CD4(+) T(H)2 memory/effector cells that produce inflammatory IL-17 cytokine and promote the exacerbation of chronic allergic asthma. J Exp Med (2010) 207(11):2479–91. doi: 10.1084/jem.20101376 PMC2964570 20921287

[52] Hegazy AN Peine M Helmstetter C Panse I Fröhlich A Bergthaler A. Interferons direct Th2 cell reprogramming to generate a stable GATA-3+T-bet+ cell subset with combined Th2 and Th1 cell functions. Immunity (2010) 32(1):116–28. doi: 10.1016/j.immuni.2009.12.004 20079668

[53] Berker M Frank LJ Gessner AL Grassl N Holtermann AV Hoppner S. Allergies - a T cells perspective in the era beyond the TH1/TH2 paradigm. Clin Immunol (2017) 174:73–83. doi: 10.1016/j.clim.2016.11.001 27847316

[54] Hayashida S Uchi H Moroi Y Furue M. Decrease in circulating Th17 cells correlates with increased levels of CCL17, IgE and eosinophils in atopic dermatitis. J Dermatol Sci (2011) 61(3):180–6. doi: 10.1016/j.jdermsci.2010.10.013 21255981

[55] Szegedi K Kremer AE Kezic S Teunissen MB Bos JD Luiten RM. Increased frequencies of IL-31-producing T cells are found in chronic atopic dermatitis skin. Exp Dermatol (2012) 21(6):431–6. doi: 10.1111/j.1600-0625.2012.01487.x 22621183

[56] Esaki H Brunner PM Renert-Yuval Y Czarnowicki T Huynh T Tran G. Early-onset pediatric atopic dermatitis is TH2 but also TH17 polarized in skin. J Allergy Clin Immunol (2016) 138(6):1639–51. doi: 10.1016/j.jaci.2016.07.013 27671162

[57] Nakahara T Kido-Nakahara M Ohno F Ulzii D Chiba T Tsuji G. The pruritogenic mediator endothelin-1 shifts the dendritic cell-t-cell response toward Th17/Th1 polarization. Allergy (2018) 73(2):511–5. doi: 10.1111/all.13322 28960333

[58] Facco M Cabrelle A Teramo A Olivieri V Gnoato M Teolato S. Sarcoidosis is a Th1/Th17 multisystem disorder. Thorax. (2011) 66(2):144–50. doi: 10.1136/thx.2010.140319 21139119

[59] Jain A Singh H Nath A Chaturvedi S Ajmani S Misra DP. Distinct T-cell immunophenotypic signature in a subset of sarcoidosis patients with arthritis. J R Coll Physicians Edinb (2020) 50(3):226–32. doi: 10.4997/JRCPE.2020.304 32936094

[60] Ramstein J Broos CE Simpson LJ Ansel KM Sun SA Ho ME. IFN-gamma-Producing T-helper 17.1 cells are increased in sarcoidosis and are more prevalent than T-helper type 1 cells. Am J Respir Crit Care Med (2016) 193(11):1281–91. doi: 10.1164/rccm.201507-1499OC PMC4910899 26649486

[61] Broos CE Koth LL van Nimwegen M In 't Veen J Paulissen SMJ van Hamburg JP. Increased T-helper 17.1 cells in sarcoidosis mediastinal lymph nodes. Eur Respir J (2018) 51(3):1701124. doi: 10.1183/13993003.01124-2017 29449421

[62] Miedema JR Kaiser Y Broos CE Wijsenbeek MS Grunewald J Kool M. Th17-lineage cells in pulmonary sarcoidosis and lofgren's syndrome: Friend or foe? J Autoimmun (2018) 87:82–96. doi: 10.1016/j.jaut.2017.12.012 29310925

[63] Arger NK Machiraju S Allen IE Woodruff PG Koth LL. T-Bet expression in peripheral Th17.0 cells is associated with pulmonary function changes in sarcoidosis. Front Immunol (2020) 11:1129. doi: 10.3389/fimmu.2020.01129 32774332 PMC7387715

[64] Lomax AJ McGuire HM McNeil C Choi CJ Hersey P Karikios D. Immunotherapy-induced sarcoidosis in patients with melanoma treated with PD-1 checkpoint inhibitors: Case series and immunophenotypic analysis. Int J Rheum Dis (2017) 20(9):1277–85. doi: 10.1111/1756-185X.13076 28480561

[65] Judson MA Marchell RM Mascelli M Piantone A Barnathan ES Petty KJ. Molecular profiling and gene expression analysis in cutaneous sarcoidosis: the role of interleukin-12, interleukin-23, and the T-helper 17 pathway. J Am Acad Dermatol (2012) 66(6):901–10, 10 e1-2. doi: 10.1016/j.jaad.2011.06.017 21924794

[66] Alunno A Bartoloni E Bistoni O Nocentini G Ronchetti S Caterbi S. Balance between regulatory T and Th17 cells in systemic lupus erythematosus: the old and the new. Clin Dev Immunol (2012) 2012:823085. doi: 10.1155/2012/823085 22761634 PMC3386568

[67] Zhong W Jiang Z Wu J Jiang Y Zhao L. CCR6(+) Th cell distribution differentiates systemic lupus erythematosus patients based on anti-dsDNA antibody status. PeerJ (2018) 6:e4294. doi: 10.7717/peerj.4294 29441231 PMC5808313

[68] Xing X Li A Tan H Zhou Y. IFN-gamma(+) IL-17(+) Th17 cells regulate fibrosis through secreting IL-21 in systemic scleroderma. J Cell Mol Med (2020) 24(23):13600–8. doi: 10.1111/jcmm.15266 PMC7753990 33157566

[69] Nikolic B Lee S Bronson RT Grusby MJ Sykes M. Th1 and Th2 mediate acute graft-versus-host disease, each with distinct end-organ targets. J Clin Invest (2000) 105(9):1289–98. doi: 10.1172/JCI7894 PMC315439 10792004

[70] Chasset F Le Buanec H Sicre de Fontbrune F de Masson A Rivet J Bergeron A. Evidence of Th1, Th17 and Tc17 cells in psoriasiform chronic graft-versus-host disease. Exp Dermatol (2016) 25(1):64–5. doi: 10.1111/exd.12857 26343416

[71] Fujiwara H Maeda Y Kobayashi K Nishimori H Matsuoka K Fujii N. Programmed death-1 pathway in host tissues ameliorates Th17/Th1-mediated experimental chronic graft-versus-host disease. J Immunol (2014) 193(5):2565–73. doi: 10.4049/jimmunol.1400954 25080485

[72] Nishimori H Maeda Y Teshima T Sugiyama H Kobayashi K Yamasuji Y. Synthetic retinoid Am80 ameliorates chronic graft-versus-host disease by down-regulating Th1 and Th17. Blood (2012) 119(1):285–95. doi: 10.1182/blood-2011-01-332478 22077062

[73] Frieder J Kivelevitch D Menter A. Secukinumab: a review of the anti-IL-17A biologic for the treatment of psoriasis. Ther Adv Chronic Dis (2018) 9(1):5–21. doi: 10.1177/2040622317738910 29344327 PMC5761942

[74] Cosmi L Maggi L Santarlasci V Liotta F Annunziato F. T Helper cells plasticity in inflammation. Cytometry A (2014) 85(1):36–42. doi: 10.1002/cyto.a.22348 24009159

[75] Basdeo SA Cluxton D Sulaimani J Moran B Canavan M Orr C. Ex-Th17 (Nonclassical Th1) cells are functionally distinct from classical Th1 and Th17 cells and are not constrained by regulatory T cells. J Immunol (2017) 198(6):2249–59. doi: 10.4049/jimmunol.1600737 28167631

[76] Tsanaktsi A Solomou EE Liossis SC. Th1/17 cells, a subset of Th17 cells, are expanded in patients with active systemic lupus erythematosus. Clin Immunol (2018) 195:101–6. doi: 10.1016/j.clim.2018.08.005 30118866

